# Selenium Accumulation in Unicellular Green Alga *Chlorella vulgaris* and Its Effects on Antioxidant Enzymes and Content of Photosynthetic Pigments

**DOI:** 10.1371/journal.pone.0112270

**Published:** 2014-11-06

**Authors:** Xian Sun, Yu Zhong, Zhi Huang, Yufeng Yang

**Affiliations:** 1 Institute of Hydrobiology, Jinan University, Guangzhou, 510632, P.R. China; 2 Key Laboratory of Aquatic Eutrophication and Control of Harmful Algal Blooms, Guangdong Higher Education Institutes, Guangzhou, 510632, P.R. China; 3 Department of Biotechnology, Jinan University, Guangzhou, 510632, P.R. China; Auburn University, United States of America

## Abstract

The aim of the present study was to investigate selenite effects in the unicellular green algae *Chlorella vulgaris* as a primary producer and the relationship with intracellular bioaccumulation. The effects of selenite were evaluated by measuring the effect of different selenite concentrations on algal growth during a 144 h exposure period. It was found that lower Se concentrations (≤75 mg L^−1^) positively promoted *C. vulgaris* growth and acted as antioxidant by inhibiting lipid peroxidation (LPO) and intracellular reactive oxygen species (ROS). The antioxidative effect was associated with an increase in guaiacol peroxidase (GPX), catalase (CAT), superoxide dismutase (SOD) and photosynthetic pigments. Meanwhile, significant increase in the cell growth rate and organic Se content was also detected in the algae. In contrast, these changes were opposite in *C. vulgaris* exposed to Se higher than 100 mg L^−1^. The antioxidation and toxicity appeared to be correlated to Se bioaccumulation, which suggests the appropriate concentration of Se in the media accumulation of *C. vulgaris* should be 75 mg L^−1^. Taken together, *C. vulgaris* possesses tolerance to Se, and Se-Chlorella could be developed as antioxidative food for aquaculture and human health.

## Introduction

Selenium (Se) is a natural trace element that acts either as an essential micro-nutrient or as a toxic compound in a dose-dependent manner. At low levels, it shows anti-carcinogenic effects [1], [2], [3] on mammalian development [4] and immune function [5] as well as slowing aging [6]. In contrast, high concentrations can cause the generation of reactive oxygen species (ROS), which induce DNA oxidation, DNA double-strand breaks and cell death [7].

Se in the aquatic environment comes from both natural and anthropogenic sources, such as irrigation of agricultural lands, coal mining and combustion. Typical concentrations in freshwaters are in the range of 0.13–2.50 nmol L^−1^ (equivalent to 0.01–0.5 µg Se L^−1^), but higher concentrations reaching 5 µmol L^−1^ (equivalent to 400 µg Se L^−1^) have been observed in contaminated areas [8]. Se enters freshwaters primarily as selenite (SeO_3_^2−^) and selenate (SeO_4_^2−^). Organic selenides (Se [−II], e.g. Se amino acids, Se proteins, methylselenides), produced by biological reduction of selenite, usually occur at lower concentrations in water than inorganic Se species [9].

In aquatic ecosystems, microalgae act as a major vector of Se from water to filter-feeders and other consumers. They accumulate Se from water-column and partially transform it into organic Se before it is transferred by ingestion to higher organisms [9]. Most plant species accumulate less than 25 µg Se g^−1^ dry weight (DW) both in terrestial and aquatic natural environment and cannot tolerate higher Se concentrations; these are termed nonaccumulators [10]. In contrast, some microalgae species (such as *Spirulina platensis*) can accumulate Se at as high a concentration as 400 µg Se g^−1^ dry weight in their cells [11]. Recently, Doušková et al. [12] reported about a new selenite resistant strain of *Scenedesmus quadricauda* for Se-enriched microalgal biomass production in photosynthetic mode of cultivation. The selenium effect on the green alga *S. quadricauda* was dose-dependent and the form of the element was also crucial [13].

In algae, the essentiality of Se has been studied mainly in marine species. Selenite bioaccumulation by phytoplankton [14] and Se requirements of many of phytoplankton species from various taxons had been demonstrated [15]. The unicellular marine calcifying alga *Emiliania huxleyi* requires nanomolar levels of Se for growth [16]. Se is essential to many algae [17] and shown to protect them against oxidative damage [18]. Sometimes, the essentiality is difficult to assess, because Se is required at such low levels for most organisms that it is experimentally challenging to generate strong phenotypes of deficiency [19]. The window between Se requirement and toxicity is the smallest of any element [20], [21]. In some freshwater species (such as *S. platensis*), Se concentrations below 20 mg L^−1^ did not inhibit the growth [22]; however, above 500 mg L^−1^ sodium selenite was toxic to this alga [23].

The toxicity of metals to marine algae may be expressed in many ways. Among them is alteration in the cellular levels of reactive oxygen species (ROS), which can cause oxidation of proteins and nucleic acids as well as lipid peroxidation (LPO), leading further to inactive enzymes, disrupted membranes, mutations, and ultimately causing cell death [24], [25]. The defense of plant cells against the damaging effects of oxidative stress involves both enzymatic and non-enzymatic components [26]. A variety of non-enzymatic antioxidants such as carotene and atocopherol may play an important role in the cellular response to oxidative stress by reducing certain ROS [27].

The function of Se is mediated mostly by selenoproteins, to which the Se as a selenocysteine is inserted during translation [28], [29]. Selenoproteins include enzymes such as glutathione peroxidases (GPX) and proteins with unknown functions, which are involved in maintaining the cell redox potential [29]. *Chlorella zofingiensis* was found to be tolerant to concentrations of sodium selenite up to 100 mg L^−1^ for 6 days. Increasing the concentration of sodium selenite above 100 mg L^−1^ (up to 150 mg L^−1^) resulted in a toxic effect, since the culture collapsed after 4 days [30]. In *C. zofingiensis*, the accumulation of boiling-stable proteins and the increased activities of the antioxidant enzymes suggested that these compounds were involved in the mechanisms of Se tolerance [30]. However, apart from GPX, other enzymes such as superoxide dismutase (SOD) form an integral part in oxidative metabolism and function to transform the radical superoxide into H_2_O_2_ [31]. The accumulation of H_2_O_2_ in the cell is prevented by guaiacol peroxidase and catalase (GPX and CAT), reducing it to H_2_O [32]. In this respect, a stronger SOD activity has been found in sorrel plants under saline stress, which were affected by the presence or absence of Se [33]. Recently, Pedrero [34] reported that Se application diminished the malondialdehyde (MDA) content in land plant broccoli, thereby reducing oxidative stress provoked by cadmium.

To date, investigations on this subject have centered on the production of Se-enriched biomass as a health food or animal feed supplement [35], [36] and on the use of marine plants for the removal of toxic elements, including Se [37]. Interestingly, nutrient interactions between Se and other minerals can affect their retention. Se is known to protect against metal toxicity by the glutathione-mediated formation of Se-metal protein and selenide-metal complexes and their subsequent redistribution [22]. In fern, Se-treated plants showed greater arsenic uptake and noimprovement in growth [23]. High dietary or waterborne Se concentrations have been shown to decrease the concentration of copper (Cu) [38] and mercury [39] in aquatic organisms. It has been suggested that an insoluble Cu-Se compound may be formed in the stomach and intestine of fish before absorption or in the liver after absorption for subsequent excretion through the bile and this process in turn reduces the bioavailability of dietary Se [40]. The dietary Se concentrations used in fish were based on the adequate requirements of Se (0.6–0.8 mg Se kg^−1^) [36]. Hepatic GPX activity was highest in fish fed diets with *>*1.23 mg Se kg^−1^, followed by 0.79, 0.39 and 0.55 mg Se kg^−1^, and lowest in fish fed the basal diet [41]. Selenomethionine is, due to its enhanced bioavailability, essential both in biomedicine and to complement the diet of domestic animals. The enrichment of the Se resistant strains in selenomethionine could be scaled up to produce Se enriched algal biomass. Also, the selected Se resistant strains could be used for bioremediation of Se-contaminated surroundings. The mechanisms of remediation include biosorption and bioconversion. The economic feasibility of algal mass culture for toxic remediation greatly depends on the high biomass productivity [42]. *Chlorella* strains have a great potential to be a resource for toxic remediation due to its faster growth and easier cultivation. For instance, *Chlorella vulgaris* can remove 63–69% of the colour from the mono-azo dye tectilon yellow 2G by converting it to aniline [43]. The use of high rate algae ponds (HRAP) is an efficient approach in bioremediation of agro-industrial wastewaters. The system consists of shallow pond with dense algae cultures aerated with paddle wheels. Apart from removing the pollutants, the algae biomass generated is useful for high-quality animal feed. *Chlorella* grown in HRAP have been shown to be useful in treating rubber effluent and sago starch factory wastewater, respectively [44].

*Chlorella vulgaris*, a unicellular green alga represented common in freshwater. Many works have shown that *C. vulgaris* is a good carrier for Se accumulation. Till now, less was known about Se-induce oxidative stress and antioxidant response in *C. vulgaris.* In the present study, we chose *C. vulgaris* as the model organism. To monitor cellular response, we grew the alga with Se-amended medium and followed the growth rate, biomass, the organic and total amount of Se, the activity of antioxidant enzymes (SOD, CAT, GPX), contents of chlorophyll a, carotenoid and ROS and LPO levels. The objective of this study was to investigate the accumulation of Se in *C. vulgaris* and its effects on the antioxidant systems (including antioxidant enzymes and non-enzyme components), and to show that Se-Chlorella could be developed as antioxidative functional foods for aquaculture and human health.

## Materials and Methods

### Culture conditions and Se treatments

*Chlorella vulgaris* C7 was obtained from the Research Center of Hydrobiology of Jinan University (Guangzhou, China). Unialgal stock cultures were propagated and maintained in Erlenmeyer flasks containing BG11 medium (pH 6.8) under incubation conditions of 25°C, a photon flux density of 45 µmol m^−2^ s^−1^ provided by two white fluorescent tubes, and a light/dark photoperiod of 12 h. Flasks were continuously shaken at 100 rpm. The components of basal culture medium [45] are as follows: KH_2_PO_4_ 0.7 g L^−1^, K_2_HPO_4_ 0.3 g L^−1^, MgSO_4_·7H_2_O 0.3 g L^−1^, FeSO_4_·7H_2_O 3 mg L^−1^, glycine 0.1 g L^−1^, vitamin B1 0.01 mg L^−1^, A5 trace mineral solution 1 ml L^−1^.

*C. vulgaris* cultures in the late exponential growth phase were decanted into 250 ml flasks containing 100 ml of medium at 25±0.5°C and illuminated with fluorescent lights (45 µmol m^−2^ s^−1^ photon flux intensity) under a 12∶12 h light:dark photoperiod. The cultures were initiated at 6×10^7^ cells ml^−1^, shaken periodically and used in triplicate. All solutions and experimental containers were autoclaved at 121°C for 15 min. Sodium selenite (Na_2_SeO_3_) was added to the medium before inoculation at a concentration of 25, 50, 75, 100, 150, 200 mg L^−1^. Cultures grown without sodium selenite served as controls.

### Growth analysis

The dry weight of *C. vulgaris* was determined by drying the cells at 70°C in a vacuum oven until constant weight.

The algal growth was monitored spectrophotometrically (UV 530; Beckman Coulter, USA) at 685 nm (O.D. 685; i.e., optical density at 685 nm). The relative growth rate is Ln(N_t_/N_0_)/t. N_0_ is the value of O.D. at 685 nm measured at time 0, N_t_ is the value of O.D. at 685 nm measured at time t.

Cell numbers were counted under a microscope with a improved Neubauer haemacytometer.

### Determination of total and organic Se content

Total Se concentration was determined by Inductively coupled plasma – mass spectrometer (ICP-MS) [46]. Cultured after 6 days, the 100 mg dried algal sample was digested with concentrated nitric acid and H_2_O_2_ (3∶1 v/v) in a digestive stove (Qian Jian Measuring Instrument Co., Ltd., China) at 180°C for 3 h. Samples were allowed to cool, dissolved in 0.6% HNO_3_ and filtered through Whattman fulter paper. Further, before drying, the samples were observed under microscope for removing any contamination of non-algal particulates. The volume of each sample was maintained up to 10 ml with 0.6% HNO_3_ and analyzed the total Se content by ICP-MS. For inorganic Se determination, the 100 mg dried algal sample was extracted with 15% HCl and the extract was analyzed by ICP-MS directly. Organic Se concentration was calculated from the difference between the total Se concentration and the inorganic Se concentration.

An Inductively coupled plasma – mass spectrometer Agilent 7500ce (Agilent Technologies, Japan) was used for analysis of sample solutions. For quantification of Se, a standard addition method was used to eliminate matrix effects of residual carbon and other matrix elements. Se isotopes 77 and 82 were used, as these isotopes did not suffer from Ar-based spectral interferences. All data are presented as means ± S.D. of three experiments.

### Antioxidant enzymes assay

The *Chlorella vulgaris* cell suspensions were centrifuged, and then the pellets were resuspended in pre-chilled phosphate buffer (pH 7.0) and disrupted by ultrasonication. The homogenate was centrifuged at 10,000 *g* for 30 min and the supernatant was collected. The total protein content in the supernatant of homogenate extracts was determined according to the Bradford method, using bovine serum albumin as a standard [47].

The activities of antioxidant enzymes and level of lipid peroxidation in the protein extract were examined using spectrophotometric diagnostic kit (NanJing JianCheng Bio Inst, Nanjing, China). Basically, superoxide dismutase (SOD) activity was determined using the xanthine oxidase method, based on its ability to inhibit the oxidation of hydroxylamine by the xanthine–xanthine oxidase system [48]. Catalase (CAT) activity was measured according to the ammonium molybdate spectrophotometric method, based on the fact that ammonium molybdate could rapidly terminate the H_2_O_2_ degradation reaction catalysed by CAT and react with the residual H_2_O_2_ to generate a yellow complex which could be monitored by the absorbance at 405 nm [49]. Guaiacol-dependent peroxidase (GPX) activity was measured by quantifying the rate of H_2_O_2_-induced oxidation of GSH to oxidised glutathione (GSSG), catalysed by GPX. The GPX content in the supernatant was measured by reaction with dithionitrobenzoic acid (DTNB) and monitored by absorbance at 412 nm [50].

### Lipid peroxidation assay

The level of lipid peroxidation (LPO) was estimated by malondialdehyde (MDA) levels, which were measured using the spectrophotometric diagnostic kits (Nanjing Jiancheng Biotechnology Institute, China) as described by Buege [51].

### Reactive oxygen species assay

For determination of reactive oxygen species (ROS), *C. vulgaris* (OD_685_ = 0.90) were incubated for 0.5 h at 37°C in 100 mL of 5 mM 2, 7-dichlorofluoresceine diacetate (DCF; Sigma, St. Louis, MO, USA) dissolved in double distilled water. After incubation, the cells was rinsed in double distilled water, then suspended in 5 mL of 40 mM Tris HCl buffer, pH 7.0, and centrifuged at 15,000 g for 10 min. Fluorescence was determined using LS-5 spectrofluorometer at an excitation wavelength of 485 nm and an emission wavelength of 535 nm. Fluorescence values were obtained using a standard curve of DCF.

### Photosynthetic pigment extraction and analysis

Using the method of Inskeep and Bloom [52], 40 ml of each culture were collected at 10,000 g for 10 min to analyze chlorophyll a content. Carotenoids were extracted with 5 ml 100% methanol in the dark at 4°C for 1 day [53]. The extracts were centrifuged at 5,000 rpm for 10 min, and the supernatants were used to determine the contents of pigments by spectrophotometer (UV 530; Beckman Coulter, USA). The contents of phycoerythrin and phycoerythrocyanin were estimated according to Beer’s method [54].

### Statistics

All treatments and controls were performed in triplicates. The significance of the difference between mean values obtained from 3 independent experiments was determined by one-way analysis of variance (ANOVA) at 95% confidence interval by using SPSS software.

## Results

### Se accumulation and its effects on biomass and relative growth rate

**Figure 1:**
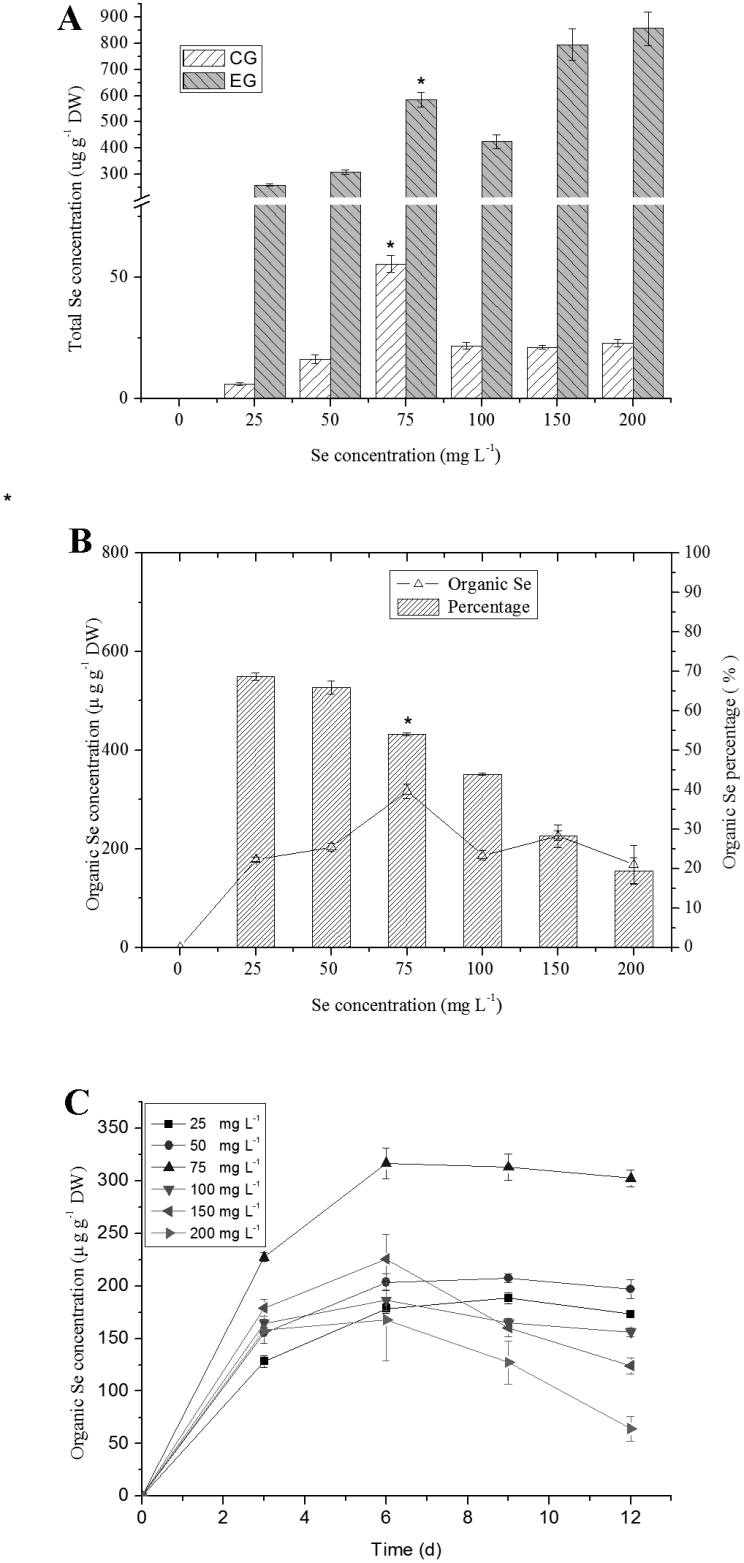
Accumulation of Se in *C. vulgaris.* (A) Total Se concentration. The control groups (CG) were spiked with 25–200 mg L^−1^ selenite within 5 minutes on the ice right before harvesting samples and the total Se content was determinated. The experiment groups (EG) were cultured at different Se concentrations for 6 days. (B) Organic Se concentration at day 6. (C) Changes in Organic Se concentration of *C. vulgaris* during cultivation time of day 3, 6, 9 and 12 at different Se concentrations. The single asterisk * in the figure represents significant difference of 75 mg L^−1^ Se treatments compared to other Se treatments (25, 50, 100, 150, 200 mg L^−1^) (*P<*0.05). Values expressed are means ± *SD* of three replicates.

In this study, *C. vulgaris* Se content increased as a function of the concentration in the medium, and biotransformed the inorganic Se to organic Se at a high rate (Fig. 1). Se content increased significantly in *C. vulgaris* as Se concentration increased from 0 to 200 mg L^−1^ (*P<*0.05). The control groups (CG) was spiked with 25–200 mg L^−1^ selenite within 5 minutes on the ice right before harvesting samples and the total Se content was determinated. Then, the total Se content of each spiked control was compared with those in the experiment groups (EG) at different Se concentrations cultured for 6 days (Fig. 1A). At low temperature, algae cells are deactivation to absorb Se into the cells within the five minutes. There is a rapid Se absorption within the first few minutes at the cell surfaces where it is irreversibly fixed and cannot be absorbed by the human body [55]. The alga cells fixed the highest Se on the surfaces at 75 mg L^−1^ Se concentration. The highest total Se content was 857 ug g^−1^ dry weight (DW). The best growth condition of the microalgae was found at 75 mg L^−1^ Se concentration. Under Se treatment of 25–200 mg L^−1^, the amount of organic Se accumulated in *C. vulgaris* increased proportionately with the cultivation time, and no lag time was observed. About 72% of organic Se was accumulated during the first 3 days of exposure. Slower increase was found from day 4 to day 6. After day 6, lower decrease was found. Maximum accumulation of organic Se was recorded at 316 ug g^−1^ under 75 mg L^−1^ Se concentration for 6 day (Fig. 1B, Fig. 1C). The Se species accumulated in *C. vulgaris* cells consisted of organic Se (*>*68%) and inorganic Se (*<*32%), showing that *C. vulgaris* was an efficient Se accumulator.

There was a direct correlation between cell numbers and the optical density at 685 nm wavelength. A regression was obtained from the data analysis.
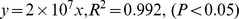



*y*: cell numbers per milliliter culture; *x*: optical density of cultures at 685 nm.

**Figure 2:**
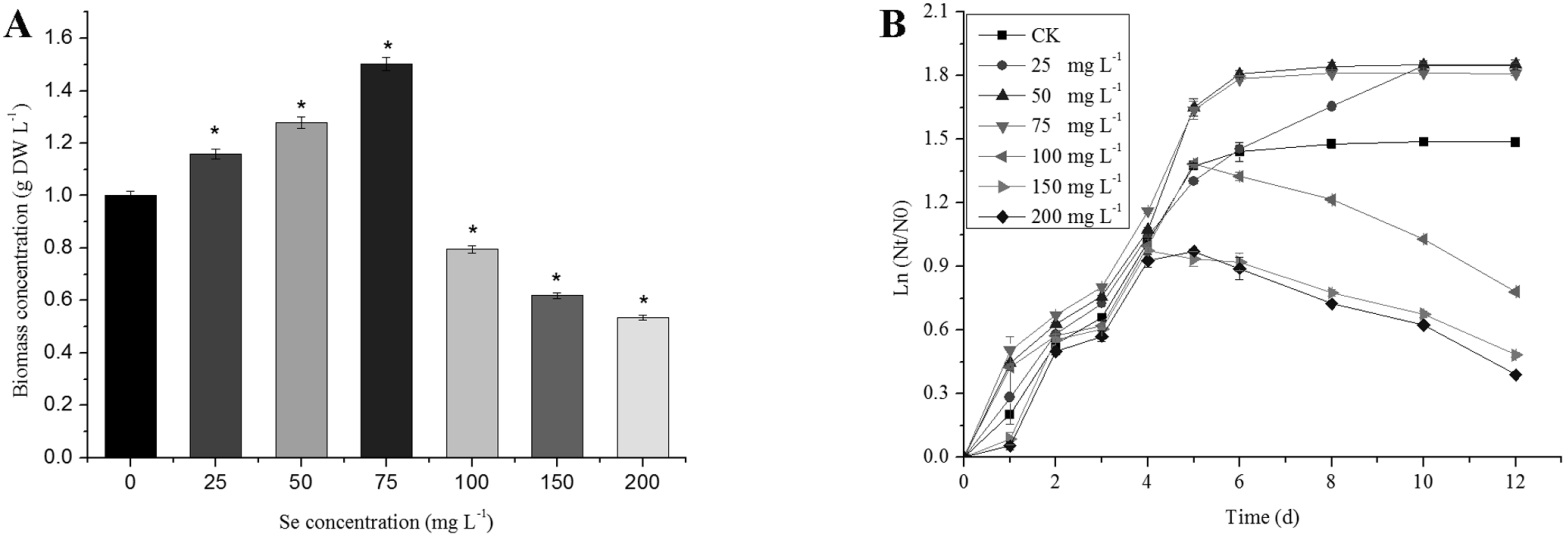
Effect of Se at different concentrations on biomass concentration (A) and relative growth rate (B). The single asterisk * in the figure represents significant difference compared to control (*P<*0.05). Values expressed are means ± *SD* of three replicates.

Based on the result, the algal growth was determined by measuring the values of OD_685_ with a spectrophotometer (UV 530; Beckman Coulter, USA) every day. Se accumulation increased the growth of *C. vulgaris* with increasing Se concentrations (25–75 mg L^−1^) significantly (*P<*0.05) and was reflected by changes in biomass concentration at the same time (Fig. 2A and 2B). The increase in biomass concentration and cell density was obtained in cells exposed to low Se concentrations (≤75 mg L^−1^), with the highest (1.5 g L^−1^ and 1.4×10^8^ cells ml^−1^) found at a Se concentration of 75 mg L^−1^ at day 6. However, higher Se concentrations (*>*75 mg L^−1^) decreased the biomass concentration and the growth of *C. vulgaris* significantly (*P<*0.05), possibly due to the toxic effects of high Se stress. Cultures turned red due to the occurrence of elemental Se when *C. vulgaris* was exposed to Se at concentrations over 75 mg L^−1^ after 6 days. By day 6, the control and 100 mg L^−1^ Se treatment groups reached the stationary phase while cultures supplemented with 25, 50 and 75 mg L^−1^ Se were still in the exponential growth phase.

### Effect of Se on the content of photosynthetic pigments in *C. vulgaris*

**Figure 3:**
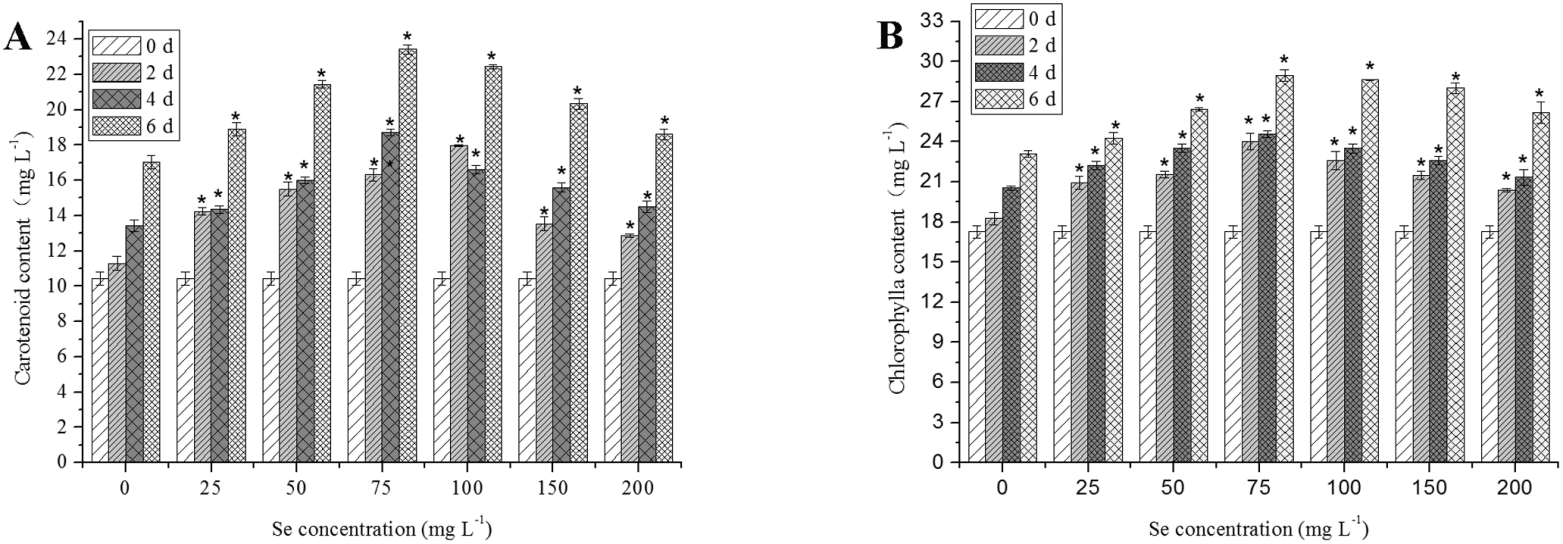
Changes in photosynthetic pigments of *C. vulgaris* during cultivation time of day 0, 2, 4 and 6 at different Se concentrations. (A) Carotenoids (including lutein and β-carotene). (B) Chlorophyll a. The single asterisk * in the figure represents significant difference compared to control (*P<*0.05). Values expressed are means ± *SD* of three replicates.

Significant (*P<*0.05) and continuous increase of carotenoid and chlorophyll a content was observed in *C. vulgaris* when it was exposed to Se concentrations up to 75 mg L^−1^. Maximum levels of carotenoid and chlorophyll a reach 23.4 and 28.9 mg L^−1^, respectively 6 days (Fig. 3). However, at higher Se concentrations (*>*75 mg L^−1^), a significant decline (*P<*0.05) in the overall content of photosynthetic pigments (including carotene and chlorophyll a) was evident when compared to the 75 mg L^−1^ Se concentrations groups.

### Effects of Se on the activities of antioxidant enzymes in *C. vulgaris*

**Figure 4:**
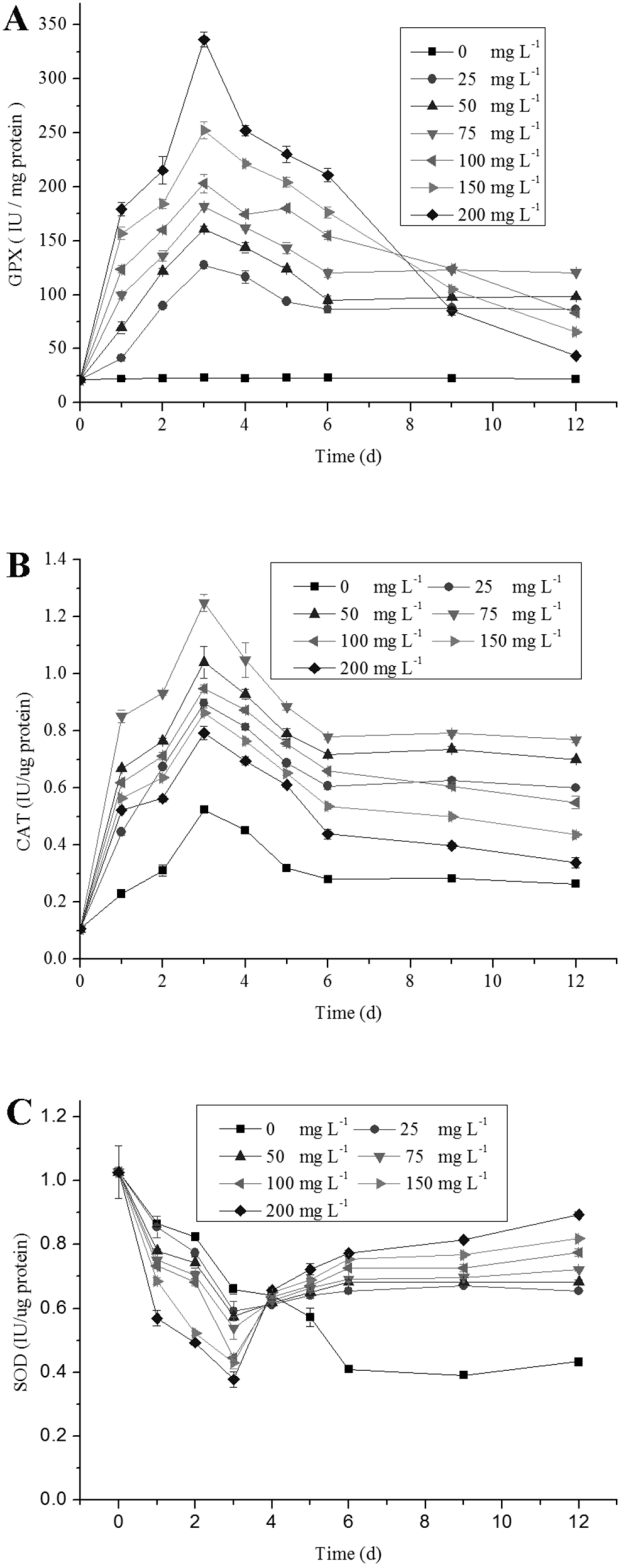
Changes in antioxidant enzyme activities of *C. vulgaris* during cultivation time at different Se concentrations. (A) GPX, (B) CAT, (C) SOD. Values expressed are means ± *SD* of three replicates.

Selenium is known to act as an antioxidant at low concentrations, but as a pro-oxidant at higher ones. In these studies, a concentration-dependent increase in activities of the antioxidant enzymes was evident since a stimulation of activity was observed under Se treatment. In comparison the control, the activities of GPX were increased when the Se concentration increased (Fig. 4A); the activities of CAT increased with increasing levels of Se from 0 to 75 mg L^−1^ (Fig. 4B). However, the control, the activities of SOD decreased when the Se concentration increased (Fig. 4C). Higher Se concentration (*>*75 mg L^−1^) inhibited the activities of CAT, compared to the low Se concentration (Fig. 4B). The maximum activities of CAT were found under the Se treatment of 75 mg L^−1^, and were 1.24±0.03 IU/ug protein. A sharp elevation in GPX activities was observed when cells were exposed to Se at 200 mg L^−1^, with maximum activity, 336±6.68 IU/mg protein, showing a positive correlation with Se concentration and GPX activity. Higher Se concentrations (100–200 mg L^−1^) resulted in significant decrease in CAT activities (*P<*0.05) compared to the lower Se concentrations (25–75 mg L^−1^), which reached their minimum (0.43±0.01 IU/ug protein) under 200 mg L^−1^ Se.

Meanwhile, time-dependent variations in the activity of antioxidant enzymes were shown in Figure 4. Compared with the control, GPX and CAT activities in cells under Se treatments both increased for up to day 3 and then significantly decreased afterwards (*P<*0.05). The maximum CAT activities were 1.24±0.03 IU/ug protein under Se treatments at day 3 (Fig. 4B). Compared with the control, SOD activity in cells under both Se treatments decreased for up to day 4 and then increased significantly afterwards (*P<*0.05) (Fig. 4C).

### Effect of Se on the intracellular ROS generation and LPO in *C. vulgaris*

**Figure 5:**
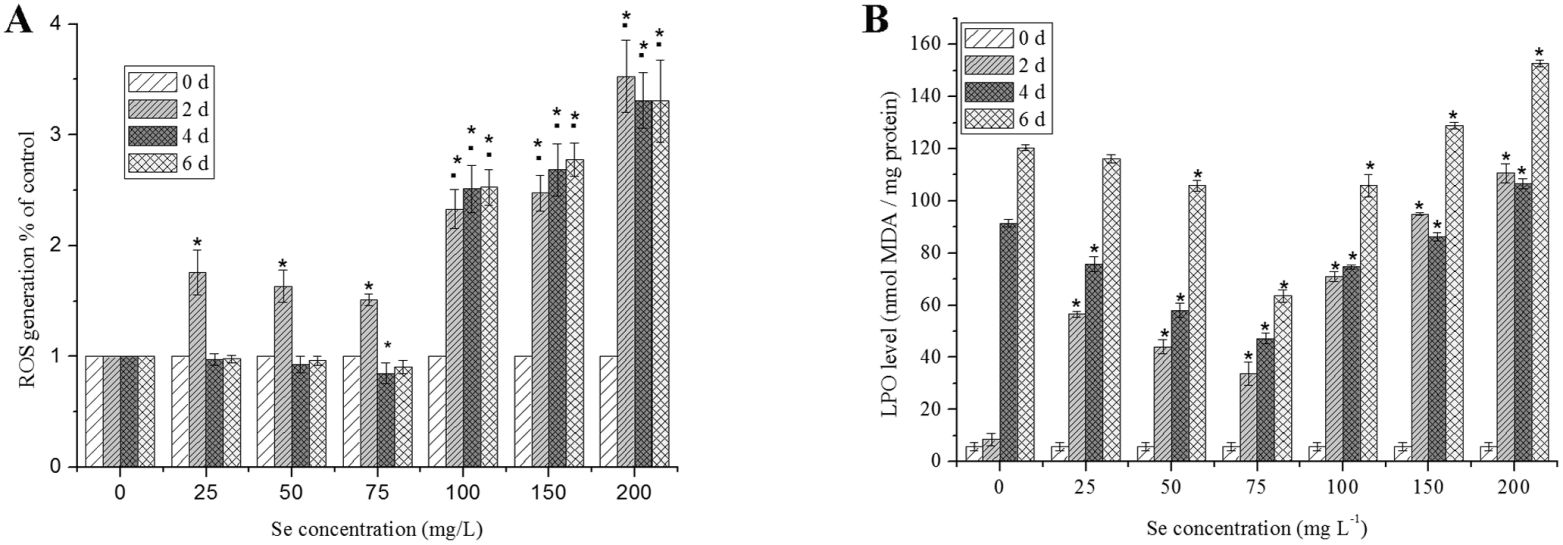
Changes in ROS (A) and LPO (B) levels of *C. vulgaris* during cultivation time of day 0, 2, 4 and 6 at different Se concentrations. The single asterisk * in the figure represents significant difference compared to control (*P<*0.05). The • in figure means significant difference of high Se treatments (100–200 mg L^−1^) compared to low Se treatments (25–75 mg L^−1^) and control (*P<*0.05). Values expressed are means ± *SD* of three replicates.

Additions of Se to culture medium ranging from 25–75 mg L^−1^, decreased the accumulation of ROS and MDA contents cultured after 2 days (Fig. 5).

The contents of ROS under the concentrations of 75 mg L^−1^ at day 4 were lower significantly (*P<*0.05) than the control (Fig. 5A). Under Se concentration of 100, 150 and 200 mg L^−1^, the content of ROS was significantly higher than the control (*P<*0.05).

LPO level decreased with the increase of Se concentrations (25–75 mg L^−1^) and reached the minimum (33.6±4.46 nmol MDA/mg protein) at 75 mg L^−1^ Se, but increased significantly (*P<*0.05) with a positive correlation to Se concentration from 75 to 200 mg L^−1^ (Fig. 5B). The maximum LPO level (152.7±1.3 nmol MDA/mg protein) was obtained at 200 mg L^−1^ Se. Significant (*P<*0.05) and continuous increase in the LPO level was observed in controls and cells under Se treatment from day 0 to day 6. However the increase trend under Se treatment of 75 mg L^−1^ was the minimum.

Cultured at day 2 under Se treatment of 25, 50 and 75 mg L^−1^, the LPO levels and ROS generation were higher than control, indicating that *C. vulgaris* was sensitive to Se initially even under low Se concentration. However, significant (*P<*0.05) decrease in the LPO level and ROS content at day 4 under Se treatment ranging from 25 to 75 mg L^−1^, showing that 75 mg L^−1^ Na_2_SeO_3_ concentration is optimum for the growth of *C. vulgaris*.

## Discussion

In green algae, Se can be transported into the cell as anionic macronutrients via a transport system but with low affinity [56]. Some algae showed growth stimulation at lower selenium levels within a defined range, but almost all were inhibited at higher concentrations [12], [57]. In this study, *C. vulgaris* showed a tolerance at high Se levels, indicating that it is a good Se bioaccumulator. However, Se has either stimulating or toxic effects on *C. vulgaris* depending on the Se concentration. According to our results, *C. vulgaris* cells can accumulate Se efficiently during cultivation at concentration ≤75 mg L^−1^. In Europe, Se levels are generally low; the main pathways for Se uptake are the selenite addition to animal feedstuffs and from seleniferous coal combustion aerosols [58]. Dietary levels of the desired amount of Se are in a very narrow range as consumption of food containing less than 100 µg Se kg^−1^ would result in its deficiency, whereas dietary levels above 1 mg Se kg^−1^ could lead to toxic manifestations [59]. At 75 mg L^−1^ with half amount of Se in inorganic form is acceptable for animal consumption. Higher Se concentrations led to much lower biomass (Fig. 2) and decreased in content of photosynthetic pigments (Fig. 3). Most of the total Se was transformed to organic Se (*>*50%) at the concentration of Se (≤75 mg L^−1^) (Fig. 1). The function of Se is mediated mostly by selenoproteins, to which the Se is inserted as a selenocysteine during translation [29]. Selenoproteins include enzymes such as GPX and proteins with unknown functions [29].

Some studies have already revealed Se is needed as a nutrient of algae in the synthesis of proteins and lipids to enhance cellular division [60]. The growth-stimulating effect of Se may be related to its antioxidative function as demonstrated by high contents of chlorophyll a (Fig. 3), and diminished LPO and ROS (Fig. 5).

The increased chlorophyll content in low Se treated cells (25–75 mg L^−1^) over control might be attributed to efficient scavenging of ROS by CAT, SOD and GPX or otherwise they would have destroyed the chlorophyll pigments [61]. Carotenoids, as one of the non-enzymatic antioxidants, play an important role in the cellular response to oxidative stress by reducing ROS [27]. Under stress conditions, singlet oxygen in the pigment bed might accumulate [62], and could seriously disrupt metabolism through oxidative damage to cellular components [63]. Carotenoids were found to be able to protect the photosynthetic membrane from photo-oxidation by effectively scavenging singlet oxygen and quenching the triplet state of chlorophyll [64]. The increase in carotenoid content in *C. vulgaris* with cultivation time was observed in both control and Se treatment cells, which may be due to these antioxidant mechanisms. However, the contents of carotenoids and chlorophyll decreased under high Se concentration (100–200 mg L^−1^), which may be due to lipid peroxidation of chloroplast membranes, resulting from cell damage or death by Se toxicity [65].

One of the Se-induced selenoproteins can protect cells from oxidative stress. A previous study showed that GPX catalyses of peroxides reduction would damage cells [66]. In this study, compared to the control, the activities of GPX increased when the Se concentration increased (Fig. 4A). SOD acts as an opposite way to GPX, indicating that lower amounts of superoxide anion radicals were produced in cells due to high GPX activity [67]. It is hypothesized that the increase in GPX, which is a scavenger of H_2_O_2_ and lipid hydroperoxides may resulted in reduced formation of superoxide anion radicals (O^2−^) through the dynamic inter-transformation among oxygen species. On the other hand, superoxide radicals can be used in spontaneous disproportion reactions producing H_2_O_2_ and singlet oxygen [68]. This supports that Se promotes through increased GPX the scavenge of produced H_2_O_2_, while it enhances the spontaneous disproportion of superoxide radicals and consequently reduces the need for their scavenger SOD (Fig. 4).

During oxidative stress, excess ROS production causes membrane damage, eventually leading to cell death. For protection against ROS, plants contain antioxidant enzymes such as SOD, CAT and GPX, as well as a wide array of non-enzymatic antioxidants [69]. ROS and LPO are the well known indices for determining the degree of oxidative stress and considered main contributors for growth retardation [70]. Se acts as an antioxidant at low concentrations. In this study, it was observed that GPX and CAT activity increased significantly and LPO level decreased significantly in cells treated with increasing levels of Se from 0 to 75 mg L^−1^ (Figure 4 and 5). However, high Se concentration may cause oxidative stress and membrane damage through production of ROS in *C. vulgaris* (Fig. 4). Compared to the low Se concentration group (25–75 mg L^−1^), the activities of CAT and GPX decreased, while the LPO and ROS levels increased under the high Se concentration (>75 mg L^−1^). From the first to the third exposure day, in spite of the decrease in SOD activity, the GPX activitiy and carotenoid concentrations increased significantly (*P<*0.05).

## Conclusion

The study provides a new insight into the impact of Se on green algae *C. vulgaris* and effects of Se on antioxidant system and bioaccumulation. Se positively promoted *C. vulgaris* growth at lower concentrations (≤75 mg L^−1^), acting as an antioxidant through the inhibition of lipid peroxidation (LPO) and intracellular reactive oxygen species (ROS). The antioxidative effect was associated with an increase in guaiacol peroxidase (GPX), catalase (CAT), superoxide dismutase (SOD) and photosynthetic pigments. Meanwhile, significant increase in the cell growth rate and organic Se content was also detected in the algae. Se is an essential micronutrient in the diet of many organisms, including humans and significant health benefits have been attributed to it. Due to its enhanced bioavailability, Se is essential both in biomedicine and complementary diet of domestic animals. The Se-*Chlorella* may be developed as antioxidative functional foods for aquaculture and human health.
